# Self Calibration of a Sonar–Vision System for Underwater Vehicles: A New Method and a Dataset

**DOI:** 10.3390/s23031700

**Published:** 2023-02-03

**Authors:** Nicolas Pecheux, Vincent Creuze, Frédéric Comby, Olivier Tempier

**Affiliations:** LIRMM, Univ. Montpellier, CNRS, Montpellier, France

**Keywords:** calibration, multibeam imaging sonar, monocular camera, dataset

## Abstract

Monocular cameras and multibeam imaging sonars are common sensors of Unmanned Underwater Vehicles (UUV). In this paper, we propose a new method for calibrating a hybrid sonar–vision system. This method is based on motion comparisons between both images and allows us to compute the transformation matrix between the camera and the sonar and to estimate the camera’s focal length. The main advantage of our method lies in performing the calibration without any specific calibration pattern, while most other existing methods use physical targets. In this paper, we also propose a new sonar–vision dataset and use it to prove the validity of our calibration method.

## 1. Introduction

Remotely Operated Vehicles (ROVs) are used for a wide range of underwater operations either physically impossible or technically complicated for divers, from inspections of industrial offshore structures to scientific deep-sea explorations. Usually, ROVs are equipped with at least one monocular video camera to pilot the ROV and to observe its surroundings. For more autonomous robots, this camera can be used for navigation by determining the robot’s position from the observed objects and features, for obstacle avoidance by tracking objects in the camera and determining the risk and time for the robot to encounter them, or even for autonomous docking using visual targets. Another example of such applications is station-keeping, which gives the ROV increased stability when standing still during inspections. This can be achieved by using homography to estimate the movement of the robot and then compensate for it [1]. Furthermore, object detection algorithms can help guide the pilot to its goal. This can be achieved using object segmentation, as presented in [2], by combining multiple visual cues (gradient, colour disparity, pixel intensity, etc.). However, all these methods are limited by optical cameras’ sensitivity to low-light conditions, colour degradation, turbidity, and noise. To cope with these problems, many techniques have been proposed to enhance underwater images, as presented in the survey [3,4]. There are also solutions to denoise underwater images using a variation of the wavelet transform [5]. Some of these algorithms are quite simple and can even be used for low-power platforms [6], such as for Autonomous Underwater Vehicles (AUVs).

**Figure 1:**
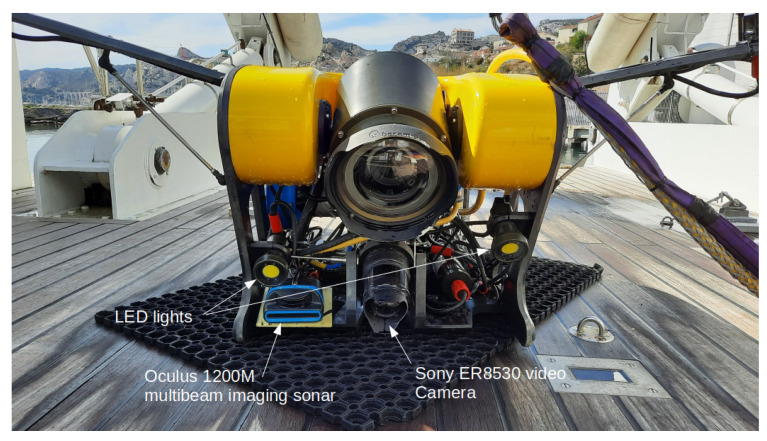
The Hilarion ROV of the DRASSM equipped with an acoustic camera (Oculus 1200M multibeam sonar from BluePrint Subsea) and a monocular video camera (Sony ER8530).

In addition to the camera, an imaging sonar may be added for specific operations (inspections of underwater structures, target localisation, etc.). An example of an ROV equipped with such sensors is shown in Figure 1. The imaging sonar allows to detect objects at a larger range or under poor visibility conditions. Moreover, sonars allow to obtain information regarding dimension and distances, which is not the case of monocular cameras. These advantages of the sonars over the cameras are counterbalanced by two limitations: a slower frame rate, due to the sound propagation; a poorer resolution, due to the limited number of acoustic beams and the quite low frequency of the emitted acoustic waves (typically less than 1.2 MHz). There are several classes of sonars. In this paper, we will only consider multibeam imaging sonars, often called “acoustic cameras”. Unlike single-beam scanning sonars, multibeam imaging sonars use several beams at the same time (typically 256), allowing a much higher update rate (typically 10 to 30 fps, depending on the range). The acoustic beams have a quite large vertical aperture (typically 20°) while having a narrow horizontal width (less than 1°). Thanks to their long range and their ability to work in turbid waters, sonars are very useful for underwater object or landmark detection and recognition. In [7], this is achieved by processing the beams composing the sonar image and by looking for combined bright spots and acoustic shadows in the acoustic image; then, comparing the sizes of the detected bright and shadow zones to a known template of the landmark leads to its recognition. These landmarks are then used for the localisation of Autonomous Underwater Vehicles (AUVs). Another use of sonar imaging is marine life detection for ecological surveys [8] using machine learning algorithms such as k-nearest neighbours, support vector machines, and random forests. To classify them, the detected targets are described using many parameters, such as their size, intensity, speed, time in the image, or time of the observation. Sonars can also be used to detect dangerous objects. For example, in [9], the authors used a CNN-based approach to identify underwater mines lying on the seafloor. In pipeline following and inspection, sonars are also often employed. A recent approach used a constant false alarm algorithm to extract the pipeline in spite of the noise in the sonar image [10].

**Figure 2:**
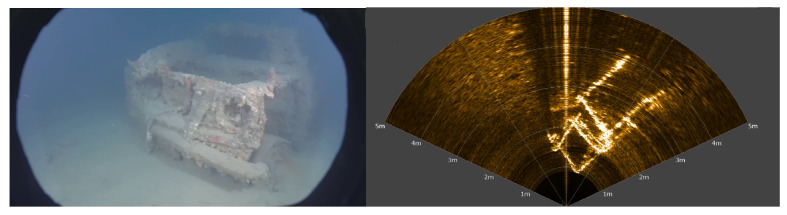
Optical image of a car acquired by a UHD (4K) camera (on the left) and the corresponding acoustic image obtained by a multibeam imaging sonar (on the right). One can observe the bright lines corresponding to the edges of the wreck.

Combining the sonar with a monocular camera allows to benefit from both sensors’ advantages: long range sensing, distance and dimension measurements, robustness to turbidity in the sonar image, easier identification of objects in the optical images, etc. Figure 2 shows acquisitions of the same scene by a video camera and sonar. However, this requires knowledge of the transformation matrix between the two sensors, thus allowing to match pixels of the sonar image with pixels of the optical image. Furthermore, the knowledge of this matrix allows to improve piloting experience. Indeed, areas of the optical image can be highlighted where obstacles or objects of interest are detected by the sonar.

In this paper, we propose to study an acquisition system associating a monocular camera and a multibeam imaging sonar. As mentioned above, to adequately exploit such a system, it is necessary to perform a calibration, i.e., to determine the existing transformation between the two sensors. Most existing calibration methods rely on purpose-made physical calibration patterns, which contain both optical patterns (such as checkers or aruco markers) and acoustically detectable patterns (made of materials with different textures or different backscattering properties). For example, in [11,12], the authors use a grid where the edges create bright lines, which intersect at corners, creating eligible feature points in both acoustic and optical images. Corners in both images are associated to their known positions in the grid. With enough points, it is then possible to find the transformation matrix linking the two sets of points by using the Levenberg–Marquardt algorithm. This process is quite similar to the one used for the calibration of standard optical stereovision systems. More recently, a paper proposed to use patterns such as aruco markers with metal rods [13] or bolts [14], allowing differences in sound reflection. These differences lead to bright spots where the material is highly reflective and dark spots where it is not. This creates patterns visible in both the optical and the acoustic images. Another approach consists in using a known 3D object, including an optical pattern such as a chessboard pattern [15]. By comparing the acoustic view of the object and the image of the optical pattern, it is possible to find the transformation between the two sensors.

There are other hybrid sensors’ associations for underwater perception. One of these methods uses a stereo camera placed alongside a sonar [16]. This adds the distance information to the visual data, thus allowing to match them with the distances from the sonar image. Another method, combining a monocular camera and an acoustic sensor, uses an echosounder instead of a sonar [17]. While not giving an acoustic image of the scene, this gives a distance map that can be overlapped with the optical image. Additionally, an original idea came from using a multidirectional microphone array [18]. This kind of sensor proposes the idea of using multiple microphones placed at various positions. This could be advantageous when the payload of the vehicle is limited.

As seen previously, most calibration methods between a sonar and an optical camera rely on a specific calibration object with features that can be detected and matched in both the acoustic and the optical images. These approaches are efficient, but their use at sea may be limited by some difficulties, such as the sea state or the requirement of divers and the time needed to immerse the object and to calibrate the system, especially from large vessels or offshore structures. A pre-calibration in a pool or in a harbour is not always enough, as the ROV maintenance teams often modify the system on the field to adapt it to various types of missions (pipeline inspection, hull inspection, manipulation, etc.) or simply because the maintenance implies frequent disassembly and reassembly of the robot, thus inducing small changes in the relative positions of the sensors. In this context and at the moment, we have found only one team who proposed a targetless calibration method. This approach is based on natural contours [19] and uses the fact that not only can edges be easily detected in optical images but they also create detectable bright lines in the acoustic images. Using these contours, the article proposes to match segmented images of the two sensors in order to perform the calibration. As for target-based approaches, this method may be limited by the field constraints because many underwater environments do not offer the adequate natural shapes and textures (i.e., allowing easy matching of optical and acoustic contours).

In this paper, we propose a new calibration method, using only very common underwater elements (rock, underwater structures, wrecks, etc.) without requiring any specific shape. Thus, our self-calibration technique is dedicated to hybrid sensing systems composed of a monocular camera and a multibeam imaging sonar. Unlike most existing methods, this technique does not require any artificial calibration pattern, and uses only elements of the observed scene without necessitating any knowledge about them. Our method first extracts acoustic feature points in the sonar image and tracks them with optical flow to compute their motion in two consecutive sonar frames. Then, a comprehensive search algorithm estimates the best transformation matrix by projecting these motions onto the optical image and by comparing the motions predicted from the acoustic image with the motion actually observed in the optical images. The proposed method also allows to estimate the focal length of the optical camera and, thus, does not require any prior knowledge of its intrinsic matrix. This method is validated by experiments on field data gathered during archaeological surveys. The results presented highlight the ability of the method to estimate the focal length of the monocular camera, as well as the transformation matrix between the two sensors. Another contribution of this paper is the introduction of a dataset. This dataset includes combined optical and sonar images acquired on archaeological underwater sites in the Mediterranean sea. The paper is organised as follows. In Section 2, we introduce the sensors’ models and the notations. Section 3 presents the calibration method. Then, the experimental performances of our algorithm are evaluated on field data and the results are presented and analysed in Section 4. This chapter also presents the content of the public dataset accompanying this paper. The conclusion gives some perspectives on future works and usage of this method.

## 2. Problem Statement, Notations and Models

### 2.1. Problem Statement

**Figure 3:**
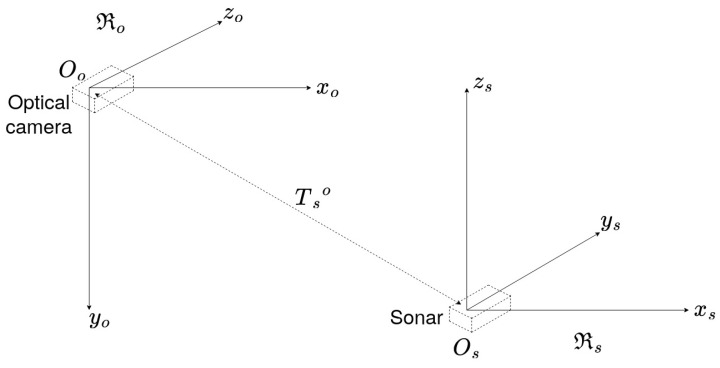
The camera frame $R_{o}$, the sonar frame $R_{s}$, and the translation vector $T_{s}^{o}$.

We consider two sensors: one monocular optical camera and one acoustic camera. Each variable associated with the monocular camera (respectively, the acoustic camera) will be referenced with a subscript *o* (respectively, *s*). Let us define $R_{s}$ as the frame associated to the sonar and $R_{o}$ as the frame associated to the optical camera as shown in Figure 3. Then, a 3D point is denoted $P_{s}:{\left(X_{s},Y_{s},Z_{s}\right)}^{⊺}$ in the sonar frame, while the same point is denoted $P_{o}:{\left(X_{o},Y_{o},Z_{o}\right)}^{⊺}$ in the optical frame. The transformation between the two frames $R_{o}$ and $R_{s}$ is composed of a 3D rotation matrix $R_{s}^{o}$ and a translation matrix $T_{s}^{o}$.

The rotation matrix $R_{s}^{o}$ is defined by three angles α, β, and γ around the axes $x_{s}$, $y_{s}$, and $z_{s}$, respectively. Using the Euler angles with the (z,y,x) convention, the rotation matrix is defined by Equation (1).

(1) $$R_{s}^{o}=R_{x}\left(\alpha \right)R_{y}\left(\beta \right)R_{z}\left(\gamma \right)=\left(\begin{array}{ccc} 1 & 0 & 0\\ 0 & cos\alpha & -sin\alpha \\ 0 & sin\alpha & cos\alpha \end{array}\right)\left(\begin{array}{ccc} cos\beta & 0 & sin\beta \\ 0 & 1 & 0\\ -sin\beta & 0 & cos\beta \end{array}\right)\left(\begin{array}{ccc} cos\gamma & -sin\gamma & 0\\ sin\gamma & cos\gamma & 0\\ 0 & 0 & 1 \end{array}\right)$$

The translation vector $T_{s}^{o}$$={\left(t_{x},t_{y},t_{z}\right)}^{⊺}$ has three components, one for each translation along the axes of the sonar frame. Then, a 3D point $P_{s}:{\left(X_{s},Y_{s},Z_{s}\right)}^{⊺}$ in the sonar frame can be expressed in the camera frame using Equation (2).

(2) $$P_{o}=R_{s}^{o}P_{s}+T_{s}^{o}$$

where $P_{o}:{\left(X_{o},Y_{o},Z_{o}\right)}^{⊺}$ are the coordinates of the 3D point $P_{o}$ in $R_{o}$, and $R_{s}^{o}$ and $T_{s}^{o}$ have been defined above and are the elements that we want to estimate through our calibration method.

### 2.2. Monocular Camera’s Model

This section details the camera model used to project a 3D point expressed in $R_{o}$ into the 2D image frame. Using the well-known pin-hole model, the projection is expressed in Equation (3).

(3) $$p_{o}=\frac{1}{Z_{o}}KP_{o}$$

where $P_{o}$ is a 3D point expressed in the camera frame; $p_{o}:{\left(u,v,1\right)}^{⊺}$ is the corresponding pixel in the optical image; and *K* is the intrinsic matrix of the camera, defined by Equation (4).

(4) $$K=\left(\begin{array}{ccc} f_{x} & s & c_{u}\\ 0 & f_{y} & c_{v}\\ 0 & 0 & 1 \end{array}\right)$$

where $\left(f_{x},f_{y}\right)$ are the focal length in pixel/m along the two axes, *s* is the skew parameter describing the non-orthogonality of pixels, and $\left(c_{u},c_{v}\right)$ are the coordinates of the optical centre of the camera expressed in pixels. For our method, we assume that the skew parameter *s* is equal to zero since it is now the case for most cameras thanks to modern manufacturing techniques (as said in [20]), and we also assume that coordinates $\left(c_{u},c_{v}\right)$ correspond to the middle of our image. Only the focal length remains unknown, with the assumption that $f_{x}$ and $f_{y}$ have the same value, noted *f*. Even though *f* can be obtained by a classic intrinsic calibration, we decided to include it in our calibration method to simplify as much as possible the calibration process to the ROV’s operator.

### 2.3. Sonar’s Projection Model

**Figure 4:**
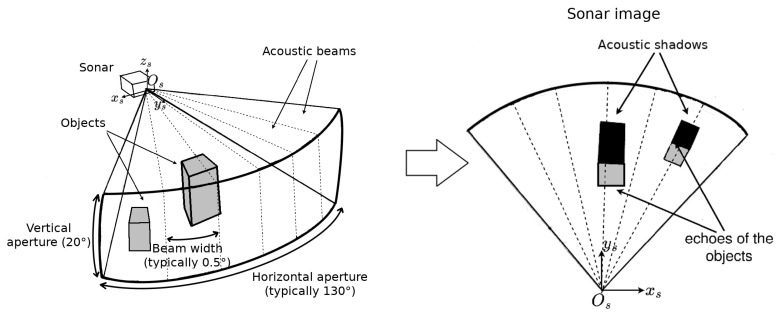
Illustration of the principle of a multibeam imaging sonar. The sensor produces wide acoustic beams, which are reflected by the objects they reach. The echoes are then received by an array of transducers forming many beams. The echoes create bright points in the acoustic image. The areas located behind the objects do not receive any sound, thus creating dark zones, corresponding to the acoustic shadows. The length of the shadow generally depends on the object’s height.

In this paper, we consider the case of a multibeam imaging sonar, which processes the echoes received along multiple beams to create an image. The principle of multibeam sonar imaging is illustrated in Figure 4.

**Figure 5:**
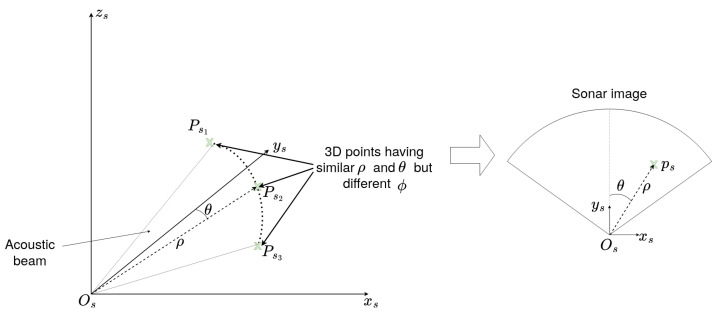
Illustration of the sonar elevation incertitude effect. In the figure, we can see that the three 3D points $P_{s_{1}}$, $P_{s_{2}}$, and $P_{s_{3}}$—having the same azimuth angles θ and the same range ρ but different elevation angles ϕ along the doted arc—will be projected on the same point $p_{s}$ in the sonar image (on the right).

In what follows, **$p_{s}$**$:{\left(\rho ,\theta \right)}^{⊺}$ will be the polar coordinates of $p_{s}$—the projection of the 3D point $P_{s}$ in the 2D sonar image $I_{s}$; ρ is the distance in meters between the sonar frame’s origin $O_{s}$ and the point $P_{s}$; while θ is the horizontal azimuth angle with respect to the central line of the sonar image (Figure 5).

As one will remark, $p_{s}$ has only two coordinates, ρ and θ, while the elevation angle ϕ does not appear. This is because the sonar cannot discriminate the echoes from points having the same horizontal azimuth and the same distance but different elevations. So, every 3D point in spherical coordinates **$P_{s_{k}}$**$:{\left(\rho ,\theta ,\phi \right)}^{⊺}$ with the same distance ρ and azimuth θ will be projected on the same point **$p_{s}$**$:{\left(\rho ,\theta \right)}^{⊺}$ of the sonar image as long as their elevation ϕ is within the range of the vertical aperture of the sonar. Figure 5 illustrates this.

This inability to discriminate the elevation angle has been studied in works concerning 3D reconstruction from sonar images. To deal with this, the existing methods either rely on a single sonar or on adding an additional sonar placed orthogonally [21] to compute the elevation angles by using the azimuth angles observed in the images from the second sonar. Another approach [22] consists in using multiple views by moving the sonar up and down and then in tracking points in these views to determine their elevation from their displacement along an acute angle of the object. The limitation of this method as it is presented by the author is that in case of smooth objects, extracting and following a feature can be difficult and can lead to errors in the elevation’s estimation. Another possibility is to consider the intensity of the points as an image of the elevation [23]. Even though the intensity of a pixel in the sonar image is linked to the echoes of each point of the arc of the acoustic beam, this method only works when used close to the ocean floor and with objects with a similar composition since backscattered intensity varies depending on the material. One last solution is to track the bright spot of an object and its shadow [24]. By combining the robot position and considering the evolution of the object’s position in time, particularly the moment when it leaves the image, this method allows to determine the elevation of certain points and the height of objects.

In our case, because of the possible absence of targets and the complexity of the environment, these method will not be used. Instead, we use an interval of elevation values [ϕ], where $\phi_{min}$ and $\phi_{max}$ (the minimum and maximum values of the interval) are defined by the sonar’s vertical aperture. Using this interval, we can find the interval of 3D points $\left[P_{s}\right]$ corresponding to each sonar point $p_{s}$ by using Equation (5).

(5) $$\left[P_{s}\right]=\left\{\begin{array}{c} X_{s}=\rho sin\left(\theta \right)cos\left(\left[\phi \right]\right)\\ Y_{s}=\rho cos\left(\theta \right)cos\left(\left[\phi \right]\right)\\ Z_{s}=\rho sin\left(\left[\phi \right]\right) \end{array}\right.$$

where the values of ϕ belong to the interval $\left[\phi_{min},\phi_{max}\right]$. Using this method means that each point in the sonar image may come from an arc of 3D points.

### 2.4. Frame Transformation

As stated before, the calibration consists in finding the parameters to go from a pixel **$p_{s}$** of the sonar image to its corresponding pixel **$p_{o}$** in the optical image. This transformation relies on the sensors’ models and the transformation between the sensors’ frames. First, starting from the sonar image point $p_{s}$, its corresponding sets of points $\left[P_{s}\right]$ can be obtained from Equation (5). Then, for the set of points $\left[P_{s}\right]$, a corresponding set of points $\left[P_{o}\right]$ is found in the optical camera frame by applying Equation (2) on each points of $\left[P_{s}\right]$. Finally, from $\left[P_{o}\right]$ and using Equation (3), the corresponding set of points $\left[p_{o}\right]$ in the optical image can be found. In summary, for a point in the sonar image $p_{s}$ with an azimuth angle θ and a range ρ, as well as a value of ϕ in the interval $\left[\phi_{min},\phi_{max}\right]$, we obtain a corresponding set $\left[p_{o}\right]$ in the optical image. This transformation is summarised by Equation (6):

(6) $$\left[p_{o}\right]=\frac{1}{Z_{o}}K\left(R_{s}^{o}\left(\begin{array}{c} \rho sin(\theta )cos(\phi )\\ \rho cos(\theta )cos(\phi )\\ \rho sin(\phi ) \end{array}\right)+T_{s}^{o}\right)$$

where ρ and θ are the coordinates of $p_{s}$ in the sonar image, ϕ is within $\left[\phi_{min},\phi_{max}\right]$, and the other variables have been introduced in previous sections. In Equation (6), we need to estimate the translation vector $T_{s}^{o}$, the rotation matrix $R_{s}^{o}$, as well as the focal length *f* of the camera (inside the camera’s intrinsic matrix *K*). In order to find these parameters, we introduce a new calibration algorithm in the following section.

## 3. Calibration Method

### 3.1. Selection of a Set of Feature Points in the Sonar Images

To compute *K*, $R_{s}^{o}$, $T_{s}^{o}$, and *f* (i.e., to calibrate the optical–acoustic system), similarly to stereovision calibration, we need to select a set of corresponding feature points in both the sonar image and the optical image.

To associate points between a camera image and a sonar image, a recent method proposes to use feature matching (SuperGlue, a feature matching method based on graph neural networks) between the optical and the style-transferred sonar image (CNN-based style transfer) [25,26]. Another optical–acoustic matching is proposed by the same research team, based on the Dense Adaptive Self-Correlation Descriptor (DASC), which provides better results than other descriptor techniques such as Scale-Invariant Feature Transform (SIFT), Binary Robust Invariant Scalable Keypoints (BRISK), and Accelerated-KAZE (A-KAZE) [27]. The goal of the authors was not to calibrate the opti-acoustic system, and one notes that rotation, translation, and scale differences between two images were corrected prior to the images’ preprocessing, thanks to the knowledge of the relative sensor’s transformation. Even if the results obtained by the matching process in [27] are impressive and very relevant, the method requires that the calibration parameters of the opti-acoustic system are known. This method also necessitates that, after style transfer, the acoustic image contains patterns relatively similar to the ones of the optical image. Although in many situations the calibration of the opti-acoustic system can be performed before the mission, for the reasons given in the introduction, we propose today a method for automatic calibration. Moreover, in natural underwater environments, it may happen that the acoustic image bears no resemblance to the optical, as depicted in Figure 2, thus reducing the effectiveness of the descriptor-based methods. For this reason, in this paper, we propose a motion-based method aiming at performing the calibration of the opti-acoustic system. Relying on the comparison of the local motion in both images (optical flow), our method does not rely on the visual similarity of the images; thus, it can work in any type of environment (except completely flat bottoms) and we do not need any artificial pattern or calibration target. This method is described below.

**Figure 6:**
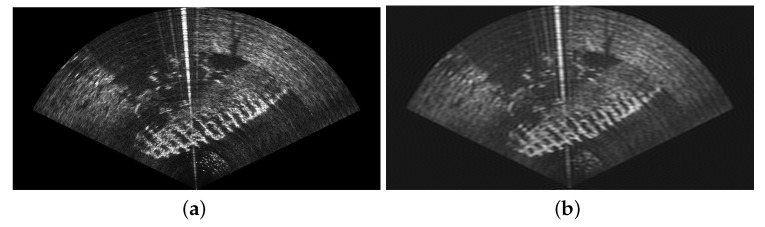
(**a**) The unfiltered sonar image. (**b**) The sonar image after using a low-pass filter.

Before selecting the points, we need to process the sonar images in order to reduce the background noise and other disturbances such as schools of fish that would appear as multiple clustered spots in the images. To suppress these, we apply a low-pass filter on the sonar image using its Fourier transform, results are shown in Figure 6. In the denoised image, denoted $I_{s_{i}}$, we select *n* feature points using the Shi-Tomasi algorithm. On sonar images, the Shi-Tomasi detector offers the advantage of selecting less outliers than the Harris detector would.

**Figure 7:**
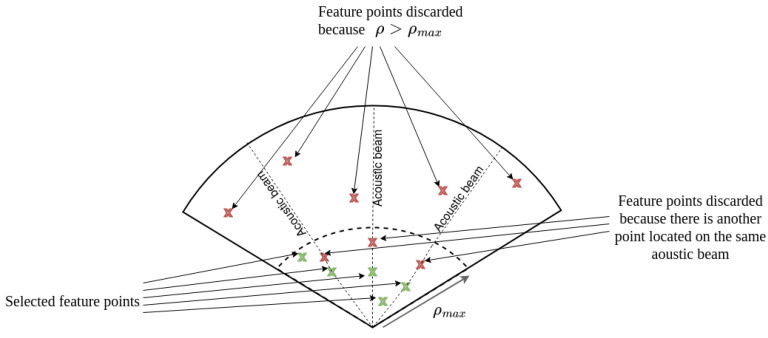
Illustration of the points’ selection process in the sonar image.

**Figure 8:**
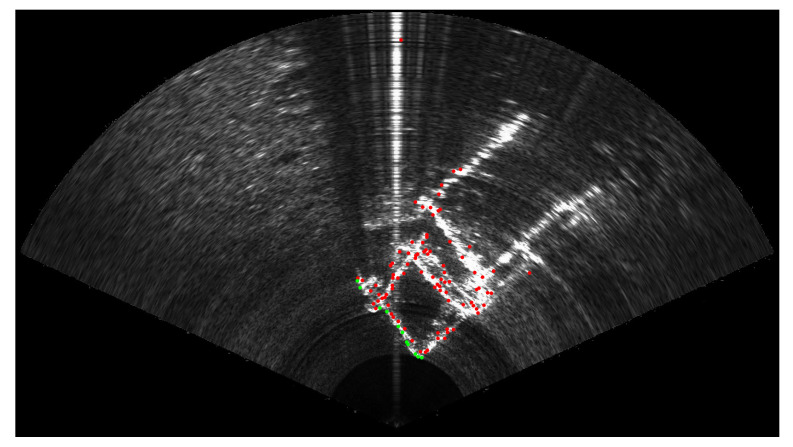
Selection process of the sonar feature points. Similarly to Figure 7, green points are the remaining points $p_{s_{i}}$ after the suppression of the red points located further than $\rho_{max}$ range or occluded by a closer point located on the same acoustic beam.

Among the *n* selected feature points in the sonar image, the ones located farther than an adjustable range $\rho_{max}$ are discarded, since they may not be visible in the optical camera due to turbidity or the lack of light (deep sea). The value of $\rho_{max}$ is set depending on the water’s turbidity and the lighting capabilities of the robot. In what follows, we will use $\rho_{max}=2$ m. We also discard the points that may be occluded by other selected points located closer on the same acoustic beam. The final set of selected points is denoted $\left\{p_{s_{i}}\right\}$ in the following. Figure 7 summarises the selection process, while Figure 8 gives an example on real sonar images. It is important to note that if the number of points in $\left\{p_{s_{i}}\right\}$ is below a certain threshold $n_{min}$, the image is discarded and the algorithm will go on to the next image. For a correct behaviour of the calibration process, experiments have demonstrated that $n_{min}$ should be equal to at least 10 points. Once a large enough set of points $\left\{p_{s_{i}}\right\}$ has been selected in the current sonar image $I_{s}{}_{i}$, these points are tracked in the next sonar image $I_{s_{i+1}}$ using the Lucas–Kanade tracking algorithm [28]. Thus, we obtain the set of sonar points $\left\{p_{s_{i+1}}\right\}$ corresponding to the tracked positions of $\left\{p_{s_{i}}\right\}$ in the second image $I_{s_{i+1}}$.

### 3.2. Projection and Evaluation

**Figure 9:**
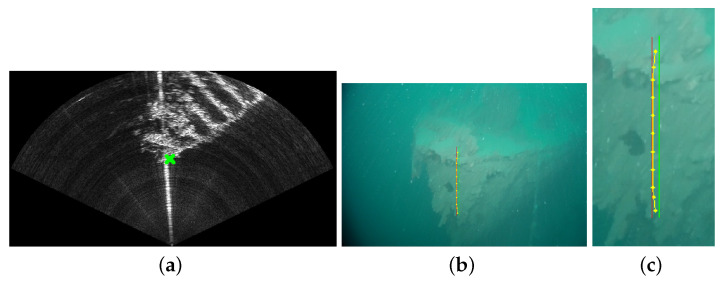
(**a**) The selected sonar point in green. (**b**) The corresponding projected points in the optical image, with the starting (green), ending (red), and estimated points (yellow); a zoomed image of the projected points is proposed in (**c**).

First, we consider an arbitrary initial value for $T_{i}$, $R_{i}$, and $f_{i}$, the sought-after parameters. Using Equations (5) and (6) presented in Section 2, we can project each starting point $p_{s_{i}}$ and the corresponding end point $p_{s_{i+1}}$ into the optical images $I_{o_{i}}$ and $I_{o_{i+1}}$ acquired at the same times $t_{i}$ and $t_{i+1}$, thus obtaining the corresponding sets of optical starting points noted $p_{o_{i}}$ and end points $p_{o_{i+1}}$. As stated before, these optical points represent an arc of points for each of the selected sonar points. We then use the Lucas–Kanade optical flow to estimate end points in the next optical image based on the optical movement of the starting points $p_{o_{i}}$, thus obtaining the estimated end points $\left\{{\hat{p}}_{o_{i+1}}\right\}$. An example of this projection process is shown in Figure 9 for a single sonar point.

So, for each selected point in the sonar image, we have a starting arc of optical points, an arc of optical end points corresponding to its tracked counterpart, and an arc of estimated points from the movement in the optical image. Using these, we can compute the projection score (i.e., a proximity score between the points computed from the optical movement and the end points obtained by projection of the tracked acoustic points).

**Figure 10:**
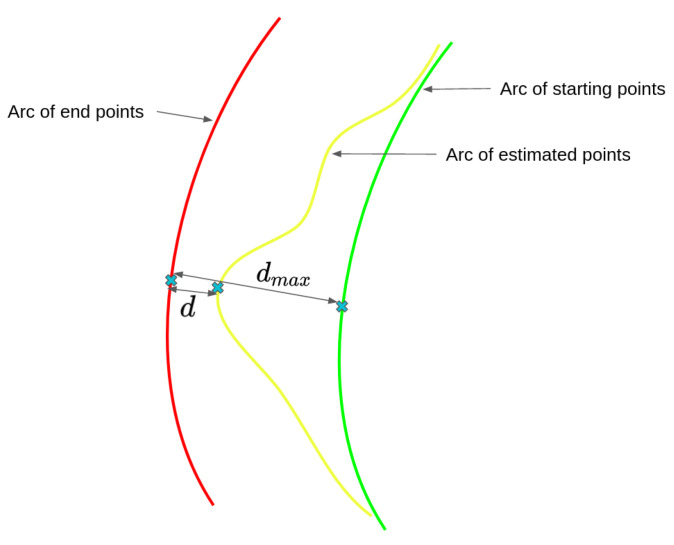
Description of the score calculation for one projected point, with the starting points (green), ending points (red), and estimated points (yellow) from the optical flow, as well as the distances used to compute the score.

The relative score for the *j-th* point is defined in Equation (7), where *d* is the minimal distance between the estimated points and the end points, and $d_{max}$ is the distance between the starting and ending points. This is illustrated in Figure 10.

This score is calculated by considering the estimated end points with the biggest displacement with respect to the starting points and their distance to the end points, noted *d*, as well as the distance between the arc of end points and the arc of starting points, noted $d_{max}$. The score for the *j-th* point among the *n* selected points is calculated by Equation (7) and described by Figure 10. We decided to represent the score with a distance ratio to mitigate the effect of parameters that could act as scale factors. We call scale factors the parameters such as focal length that will impact the scale of the projection, thus changing the spacing of the point by themselves.

(7) $$score_{j}=\frac{abs(d_{max}-d)}{d_{max}}$$

Then, by taking the mean score of every projected point, we obtain the score for one group of images (two consecutive sonar images and their corresponding optical images).

### 3.3. Estimation of the Calibration Parameters

To compute the projection parameters (i.e., calibration parameters), we iterate through all the parameters, realising an exhaustive search in a parameter space whose limits can be either defined by the dimensions of the robot or chosen by the operator according to the rough knowledge of the robot’s configuration if it is available (note that the method will work even without any prior knowledge about the geometric configuration of the setup). Since an exhaustive search can take a long time, we use an adaptive search, starting with a coarser step, and then using a finer step to find the calibration parameters. In addition, we also need to use multiple image pairs to obtain a finer estimation.

To conclude this section, all the steps of the calibration algorithm are represented in Algorithm 1.
**Algorithm 1** Research of the calibration algorithm on one set of camera and sonar image pairs.$I_{s_{i}}\leftarrow getNextSonarImage\left(\right)$$I_{o_{i}}\leftarrow getNextCameraImage\left(\right)$$I_{s_{i+1}}\leftarrow getNextSonarImage\left(\right)$$I_{o_{i+1}}\leftarrow getNextCameraImage\left(\right)$$p_{s_{i}}\leftarrow selectFeaturePoints\left(I_{s_{i}}\right)$$p_{s_{i+1}}\leftarrow LucasKannade\left(I_{s_{i}},I_{s_{i+1}},p_{s_{i}}\right)$scoreMin←+∞**for all**$R_{s}^{o}$, $T_{s}^{o}$ and *f* **do** [$p_{o_{i}}$, $p_{o_{i+1}}$] $\leftarrow projectPointsSonarToCamera(R_{s}^{o},T_{s}^{o},f,p_{s_{i}},p_{s_{i+1}})$ ${\hat{p}}_{o_{i+1}}\leftarrow LucasKannade\left(I_{o_{i}},I_{o_{i+1}},p_{o_{i}}\right)$ $projectionScore\leftarrow computeScore(p_{o_{i}},p_{o_{i+1}},{\hat{p}}_{o_{i+1}})$ **if** score<scoreMin **then**  scoreMin←score  [$T_{min}$, $R_{min}$, $f_{min}$] ← [$R_{s}^{o}$, $T_{s}^{o}$, *f*] **end if****end for****Return:** [$T_{min}$, $R_{min}$, $f_{min}$]

## 4. Experimental Validation and Dataset

### 4.1. Experimental Setup

To test our calibration algorithm, we performed two campaigns at sea with two different ROVs. These tests were performed on wrecks under the supervision of the Department of Underwater Archaeological Research (DRASSM) of the French ministry of culture. The first set of tests were performed with the *Hilarion* ROV equipped with a Sony 4K ER8530 optical camera and an Oculus 1200M multibeam imaging sonar (Figure 1).

**Figure 11:**
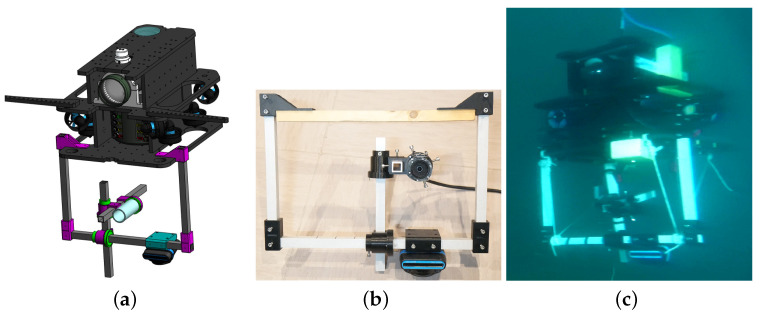
(**a**) The CAD model of the Basile ROV and its frame, allowing to modify the relative positions of the camera and the sonar. (**b**) The mechanical frame with the optical camera and the sonar. (**c**) The frame attached to the *Basile* ROV during a dive in Marseille.

*Hilarion* inspected underwater car wrecks located in the Mediterranean Sea, 60 m deep. Such wrecks are interesting for these experiments since they present sharp angles, thus facilitating the detection of feature points thanks to the bright echoes they create in the sonar images. The second set of tests were performed with the *Basile* ROV, equipped with the same Oculus 1200M multibeam imaging sonar and a monocular imaging camera, both mounted on a mechanical frame, allowing to accurately change the geometric parameters (e.g., distance and orientation of the camera with respect to the sonar) and thus allowing us to control the ground truth of the extrinsic calibration parameters, as shown in Figure 11. During this second mission, the ROV observed various wrecks (cars, barges, boats, etc.) located around 60 m deep. We created a software allowing synchronisation of the images from the two sensors, as well as the IMU of the robot.

### 4.2. Dataset

The dataset we created contains 17572 monocular images and the 8577 corresponding sonar images. We also added the IMU data of the ROV during the mission, despite them not being useful for our calibration method. We named this dataset the “shipwreck sensing dataset” and it is publicly available here https://www.lirmm.fr/shipwreck-dataset/ (accessed on 1 February 2023).

**Figure 12:**
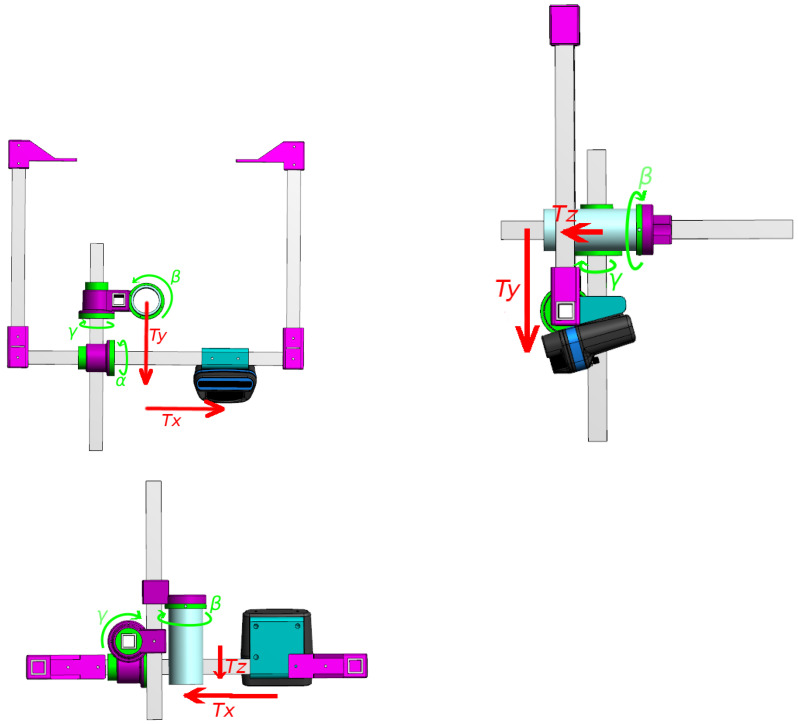
Representation of the extrinsic parameters as part of the frame used to set them during the experiments at sea. It is important to note that the rotations are expressed along the sonar frame $R_{s}$ while the translations are expressed along the optical camera frame $R_{o}$, as defined in Figure 3.

Details on the nature of the data and their acquisition are presented in Table 1. In order to see if our algorithm works for various positions of the sensors, we acquired images with different configurations, as presented in Table 2. The choice of these ground truth configurations was made to try parameters independently, the first one serving as a reference and the two others introducing variation on a single parameter. A representation of each of the extrinsic parameters is shown in Figure 12.

### 4.3. Experimental Evaluation of the Calibration Algorithm

Taking sonar and camera image pairs from this dataset, we tested our algorithm using the steps described in Section 3. The code was made in C++ with the OpenCV library and executed on a Dell precision 5520 with an Intel Xeon E3-1505M v6 3.00 GHz processor.

**Figure 13:**
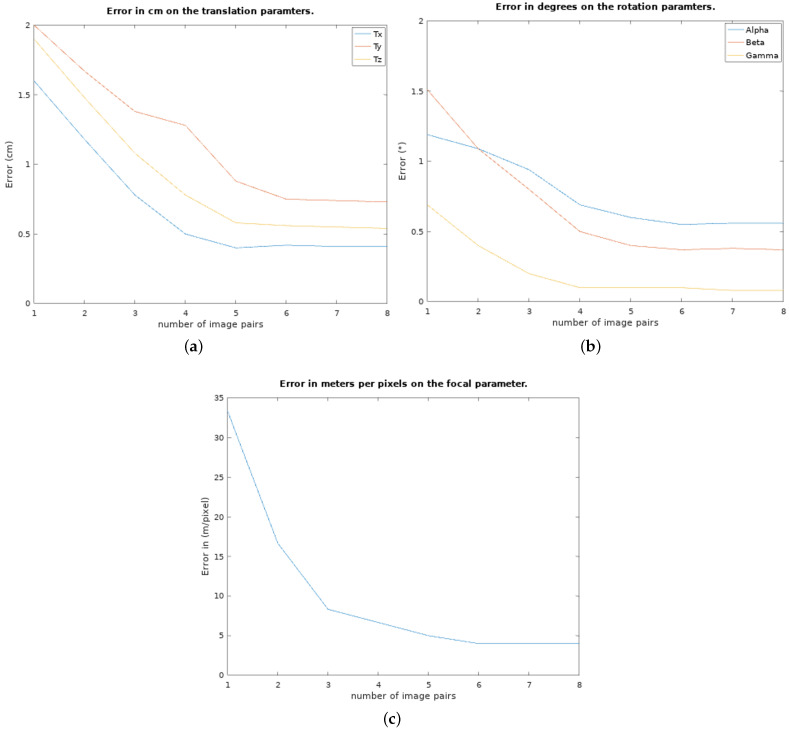
(**a**) The evolution of the mean error on the calibration parameters over the number of image pairs used. In (**a**), the translation parameters ($T_{s}$, $T_{y}$, and $T_{z}$) are presented in meters, (**b**) the rotation parameters (α, β, and γ) in degrees, and (**c**) is the focal parameter.

First, we tested our algorithm on an increasing number of image pairs to show the evolution of the error. The error is the absolute value of the difference between the parameters obtained with the algorithm and the ground truth (relative positions of the two sensors on the frame, and focal length computed from a standard optical calibration of the camera). The results are presented in Figure 13. One observes that the algorithm converges very fast (5 to 6 pairs of images) to errors smaller than 1 cm and 1 degree.

**Figure 14:**
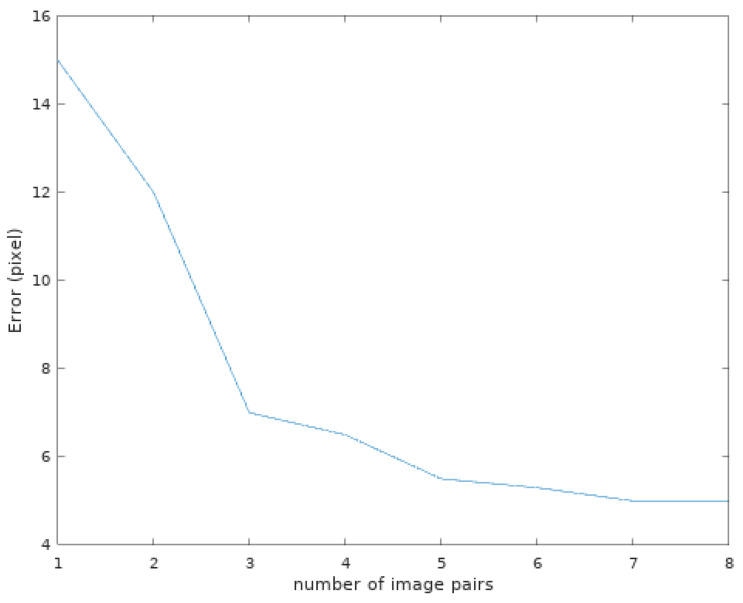
The mean reprojection error (in pixels) depending on the number of image pairs used for the calibration. As a reminder, the images have dimensions of 720 × 480 pixels.

**Figure 15:**
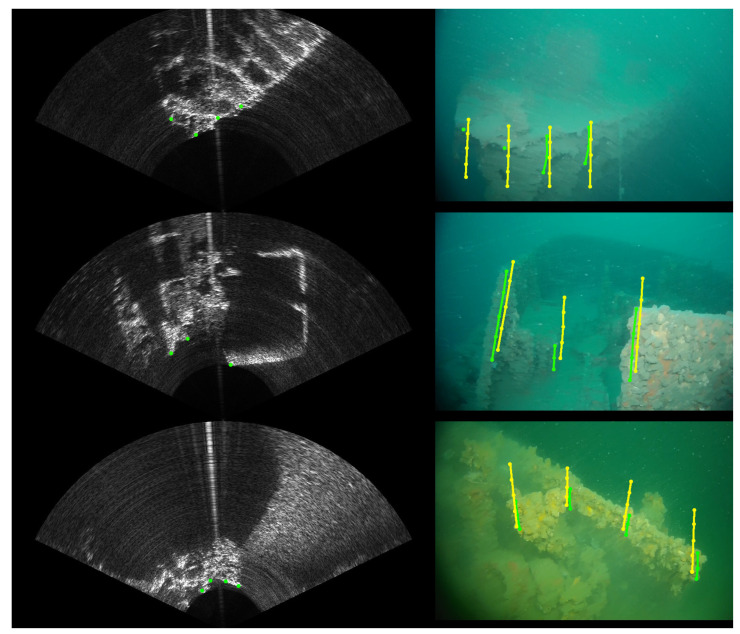
Examples of reprojection once the calibration parameters have been obtained. In green are the selected points in the sonar image and their corresponding area of presence in the camera image. In yellow are the arcs of points obtained using the found calibration parameters.

As we could expect, the results yield a bigger error on the β and $T_{z}$ parameters because of the elevation uncertainty in the sonar images, creating a larger vertical zone where the projection can match the movement. Similarly with the error on the parameters shown in Figure 13, the evolution of the reprojection error in pixels is shown in Figure 14. This reprojection error is defined by the minimal distance between the projected arcs and the known position where they should be. An example of points projected with the found calibration parameters in comparison to their goal is shown in Figure 15.

The achieved results allow to accurately convey information (the position of an object from one to the other, for example) from the sonar to the camera and vice-versa, notably for the position of objects seen by the sonar from further away. Even though a greater number of images yields a lower error, it is at the cost of the time required to obtain the results. Since this is an exhaustive search without any optimisation, the time increases with the number of image pairs (around 4 h per pair), requiring several hours to compute the calibration parameters, despite using a coarser step to reduce search time (typically searching by 5 cm/° every iteration, then reducing the step to 3, and then 1).

This steep increase of the required time can be explained by the sequential implementation of this algorithm (no parallelisation). An improvement on that matter could be a subject of future work. The purpose of this brute force approach was to validate the algorithm before improving its time of execution. To end this section, Table 3 summarises the results yielded on all the configurations available in the dataset.

These results show that we are able to achieve a precise estimation of all the parameters despite the differences in configuration. Even though an error still persists, we consider it sufficiently low for applications making these two sensors work together. For example, with such precision we could highlight in the optical image the position of a distant object visible only in the sonar image.

Table 4 presents a comparison with results from the literature. One can observe that we obtain better performances for translation estimation and we obtain 0.5 degree less accurate results for rotation estimations. This shows that using movement is an effective way to compute the calibration parameters.

The main limitation of our method in its current form is the important time required to estimate the calibration parameters. This makes our method unusable for short missions; however, it could still be of used for long-term missions. This drawback is counterbalanced by the fact that our method does not require any specific calibration pattern and can be performed in any natural environment. The computation of the parameters relies on brute force; thus, it is likely to be optimised in the future in several ways. As gradient-based techniques are likely to fail with such a problem, we will consider other approaches in the coming months, such as genetic algorithms. In addition to this, although it is not required for the convergence of the algorithm, a rough measurement of the relative positions of the two sensors with a very reasonable accuracy of several centimetres and several degrees would drastically reduce the search space and, thus, will help them to converge much faster.

## 5. Conclusions

In this article, we presented a new targetless calibration method for a system combining an acoustic camera (i.e., multibeam imaging sonar) and an optical monocular camera. This method uses the pixels’ motion in the images of the two sensors. After a presentation of the model of each sensor, we showed that we could project the movement of feature points of the sonar image into the optical image. Using the optical flow of the optical image to obtain an estimate of the movement of projected points in the optical image, a distance score was calculated, allowing us to compute the calibration parameters through an exhaustive search. The important upside of this method is that it does not require a calibration pattern. This will help for robotic operations at sea, which may require frequent recalibration due to changes in the sensors’ positions and orientations. The obtained level of accuracy is sufficient to merge the data acquired by the two sensors and is close to the one obtained by existing calibration methods based on a target. Future works will consist in optimising the algorithm to improve the search speed, with the goal of reaching a far better execution time, preferably below an hour, while keeping the same precision. For now, plans for this method are to use it to highlight in the optical image the distant structures (objects, rocks, pipelines, etc.) that are visible only to the sonar, in order to give better indication to the ROV’s operator.

## Figures and Tables

**Table 1:** Technical data about the sensors of the Basile ROV.

Monocular Video Camera	
Camera model	Optovision HD mini IP camera
Image size	720 × 480 pixels
Frame rate	30 fps
**Sonar**	
Sonar model	Oculus 1200 M
Image size	1024 × 507 pixels
Frame rate	10 fps
Horizontal aperture	130
Vertical aperture	20
Angular resolution	0.5
IMU data frequency	20 Hz

**Table 2:** The three geometric configurations of the sensors available in the dataset.

	$T_{x}$ (cm)	$T_{y}$ (cm)	$T_{z}$ (cm)	α (°)	β (°)	γ (°)	*f* (pixel/m)
Configuration I	0	5	0	0	0	0	600
Configuration II	0	15	0	0	0	0	600
Configuration III	10	5	0	0	0	0	600

**Table 3:** Results obtained with our method for the three geometric configurations.

	$T_{x}$ (cm)	$T_{y}$ (cm)	$T_{z}$ (cm)	α (°)	β (°)	γ (°)	Focal
Configuration I ground truth	0	5	0	0	0	0	600
Configuration I estimated	1.2	3.8	0.9	0.7	1.0	0.1	570
Configuration II ground truth	0	15	0	0	0	0	600
Configuration II estimated	0.5	14.2	0.8	0.3	1.1	0.4	610
Configuration III ground truth	10	5	0	0	0	0	600
Configuration III estimated	8.7	4.0	0.8	0.7	1.0	0.1	570

**Table 4:** Comparison between existing methods and our algorithm.

Algorithm	Error on $T_{x}$ (m)	Error on $T_{y}$ (m)	Error on $T_{z}$ (m)	Error on α (°)	Error on β (°)	Error on γ (°)
[12]	0.02	0.05	0.1	0.1	1.0	0.003
[14]	0.0	0.05	0.1	1.0	5.0	0.0
Our algorithm	0.01	0.015	0.05	1.0	1.5	0.5

## Data Availability

As stated in Section 4.2, all the data presented in this article and acquired in order to test this method are available at https://www.lirmm.fr/shipwreck-dataset/ (accessed on 1 February 2023). There, both optical and sonar images as well as the IMU data during the mission can be downloaded.
